# Toothpaste‐Related Attitudes and Practices of Parents of Preschool Children in Istanbul, Türkiye: A Cross‐Sectional Study

**DOI:** 10.1155/ijod/8522497

**Published:** 2026-10-30

**Authors:** Melis Unsal-Caliskaner, Zeynep Seda Uyan, Senem Selvi Kuvvetli

**Affiliations:** ^1^ Department of Paediatric Dentistry Faculty of Dentistry Yeditepe University Istanbul Türkiye yeditepe.edu.tr; ^2^ Department of Paediatric Dentistry Faculty of Dentistry Istanbul Atlas University Istanbul Türkiye; ^3^ Division of Pediatric Pulmonology Faculty of Medicine Koc University Istanbul Türkiye ku.edu.tr

**Keywords:** early childhood caries, fluoride, oral hygiene, parental attitudes, preschool children, toothpaste usage

## Abstract

**Objectives:**

To assess parental attitudes and practices regarding toothpaste usage in preschool children attending paediatric outpatient clinics in Istanbul, Türkiye, and to examine associations with socio‐demographic characteristics.

**Methods:**

A cross‐sectional questionnaire study was conducted among 203 parents of children under 6 years attending paediatric outpatient departments at two university hospitals in Istanbul (June 2022–February 2023). Toothpaste‐related items covered: whether toothpaste was used during brushing and how frequently; reasons for toothpaste usage; whether the child had a separate toothpaste; age at which toothpaste was introduced; who applied toothpaste; amount used; post‐brushing behaviour; criteria when choosing toothpaste; and reasons for brand selection. Chi‐square tests and *Z* tests with Bonferroni correction assessed associations with parent age, education, sex, number of children and child age group.

**Results:**

The majority of children (84.2%) were reported to use toothpaste during toothbrushing, while only 71.4% used it every time. Only 12.3% of parents started using toothpaste immediately after eruption of the first deciduous teeth, while 70.2% began between ages 1–3 years. More than half of parents (59.1%) put toothpaste on their children’s toothbrush themselves, and 64.9% used less than 1/3 of the brush length. However, 22.4% of children swallowed toothpaste while brushing, significantly more common in children under 2 years (61.5%) and in single‐child families (32.9% vs. 11.1%; *p* = 0.020). Consistent toothpaste usage was significantly associated with child age (*p*  < 0.001) and parent age (*p*  < 0.001). Most parents (87.1%) considered the contents of toothpaste when choosing, while only 8.8% specifically mentioned fluoride. Nearly half (47.6%) chose their toothpaste brand due to organic/herbal ingredients, and the most popular brand chosen was a fluoride‐free product (29.9%).

**Conclusions:**

Despite high overall rates of toothpaste usage, important gaps remain: delayed introduction, high rates of toothpaste swallowing among young children and critically low awareness of fluoride as a key toothpaste ingredient. Targeted parental education, particularly on the benefits of fluoridated toothpaste, appropriate quantity and correct post‐brushing behaviour, is urgently needed.

## 1. Introduction

Oral health is an integral component of general health and can affect the functional, psychological and social dimensions of well‐being [1]. Dental caries is one of the significant public health problems in the world and one of the most common chronic diseases of early childhood, with a prevalence of an estimated 621 million children with untreated caries in primary dentition [2, 3]. A nationwide oral health survey in Türkiye reported that dental caries prevalence was 69.8% for 5‐year‐old children [4].

Toothpaste is an essential element of oral hygiene. Fluoride is the most effective anti‐caries therapeutic agent and the gold standard in toothpastes [5]. A meta‐analysis by Marinho et al. [6] showed strong evidence of fluoridated toothpastes’ clinical effectiveness in preventing dental caries. Children under 6 years have inadequate swallowing and expectorating reflexes and may ingest 25%–65% of the toothpaste applied to their toothbrush. To avoid the risk of dental fluorosis, an age‐related amount of fluoridated toothpaste should be applied; parents are advised to dispense toothpaste in every use to minimise fluoride consumption [7, 8]. International guidelines, including those of the American Academy of Pediatric Dentistry [9] and the European Academy of Paediatric Dentistry [5], recommend a smear‐ or rice‐sized amount of fluoridated toothpaste for children under 2–3 years and a pea‐sized amount thereafter, alongside parental supervision of brushing. The methods used for rinsing behaviour after brushing significantly influence the anti‐caries effect of fluoride [10].

Parents and children are known to visit hospitals and family health centres earlier than dental clinics [ 11 ]. To our knowledge, there are no existing reports on parents’ toothpaste‐related attitudes and practices among families attending paediatric outpatient departments in Türkiye. The choice of toothpaste is affected by factors such as socio‐economic status, product recommendations, television and internet advertisements and social media [12, 13]. There are many different commercial brands of toothpaste for children in Türkiye. Egil et al. [14] reported that the anti‐caries effect is the most crucial reason for toothpaste preference of parents in Türkiye. By focusing on families attending paediatric outpatient departments rather than dental clinics, this study provides new insight into toothpaste‐related attitudes and practices in a broader population, complementing findings from previous studies conducted in paediatric dental clinics. Understanding parental toothpaste‐related practices is essential for designing effective oral health promotion strategies. Therefore, the aim of this study was to assess parents’ attitudes and practices regarding toothpaste usage for preschool children attending two different paediatric outpatient departments in Istanbul, Türkiye.

## 2. Methods

### 2.1. Study Design and Participants

Sample size was determined a priori using 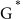
Power 3.1. Based on effect sizes reported in the literature for toothpaste type (0.09) [12] and toothbrushing frequency (0.1) [15], power analyses at alpha = 0.05 and power = 0.80 indicated minimum requirements of 192 and 158 participants, respectively; a minimum of 195 participants was therefore planned. This was a cross‐sectional questionnaire study conducted among 203 parents of children aged under 6‐year‐old, attending the Paediatric Outpatient Departments of Koç University Hospital and Yeditepe University Hospital, Istanbul, between June 2022 and February 2023. The selection of the participants was done using a random sampling technique based on the following inclusion criteria: parents of children under 6 years old, children with at least one erupted deciduous tooth and children with no mental or systemic conditions. Of 210 questionnaires collected, seven were excluded because of unanswered questions or multiple markings on single‐response items, yielding a final sample of 203 participants (104 from Koç University Hospital; 99 from Yeditepe University Hospital). Written informed consent was obtained from all participants.

### 2.2. Questionnaire

The questionnaire was developed using validated instruments from previous studies [15–17] and translated by two independent translators. The final version was cross‐examined by a content evaluation panel of 7 experts from Yeditepe University Faculty of Dentistry, Department of Paediatric Dentistry. After calculating the content validity index and content validity ratio, all questions were approved. Questionnaires were administered by a single researcher under observation. Toothpaste‐related items addressed: (i) whether the child uses toothpaste during brushing and how frequently; (ii) reasons for toothpaste usage; (iii) whether the child had a separate toothpaste; (iv) age at which toothpaste was introduced; (v) who applies toothpaste; (vi) amount used; (vii) post‐brushing behaviour; (viii) criteria when choosing toothpaste and (ix) reasons for brand selection.

### 2.3. Statistical Analysis

All collected data were analysed using IBM SPSS software Version 23. Associations between toothpaste‐related practices and socio‐demographic variables were assessed using chi‐square tests; multiple comparisons were examined using the *Z* test with Bonferroni correction. A *p*‐value of < 0.050 was considered statistically significant. Multivariable logistic regression, with Firth’s penalised regression as a sensitivity analysis, was used to assess independent predictors of toothpaste‐related practices while adjusting for potential confounders (parental age, education, number of children, child age group and child sex).

## 3. Results

The mean parental age was 36.15 ± 5.57 years; most participants were mothers (80.8%) and university/college graduates (44.8%). The mean child age was 3.66 ± 1.52 years, with 82.3% aged 2–6 years. Full demographic data are reported in the companion paper. The frequency and percentage distribution of responses to all toothpaste‐related questions are presented in Table 1.

**Table 1:** Frequency and percentage distribution of toothpaste‐related responses (*N* = 203).

Question/response	*N*	%
Does your child use toothpaste when brushing?		
Uses toothpaste every time	145	71.4
Uses toothpaste, but not every time	26	12.8
Does not use toothpaste	23	11.3
Has not started brushing yet	9	4.4
Reasons for using toothpaste (multiple response)^a^		
To prevent dental caries	123	71.9
To prevent colouring/staining of the teeth	89	52.0
As part of the daily routine	68	39.8
To prevent gum diseases	62	36.3
To provide a fresh breath	49	28.7
Other	2	1.7
Does your child have his/her own separate toothpaste?		
Yes	171	99.4
No	1	1.7
When did you start using toothpaste for your child?		
As soon as eruption of the first deciduous teeth	21	12.3
Between ages 1–3 years	120	70.2
After age 3 years	30	17.5
Who puts toothpaste on your child’s toothbrush?		
The child himself/herself	63	36.8
Mother or another adult	101	59.1
Sometimes child, sometimes adult	7	4.1
Amount of toothpaste used		
Less than 1/3 of the brush length	111	64.9
Less than 2/3 of the brush length	48	28.1
Full length of the brush	11	6.4
Other	1	0.6
Post‐brushing behaviour		
Rinses with plenty of water and spits out	111	65.3
Spits out without rinsing	21	12.4
Swallows toothpaste while brushing	38	22.4
Criteria when choosing toothpaste (multiple response)^a^		
The content of the toothpaste	148	87.1
The taste/aroma of toothpaste	28	16.5
Other (e.g., age appropriateness)	11	6.5
Images on the package	6	3.5
The price of the toothpaste	6	3.5
Reasons for brand selection (multiple response)^a^		
Organic/herbal toothpaste	81	47.6
Dentist’s recommendation	51	30.0
Information on the package	41	24.1
Accessibility	26	15.3
Recommended by friends/acquaintances	25	14.7
Aroma/taste	24	14.1
Containing fluoride	15	8.8
Price	4	2.4
Other	12	7.1

*Note:* Percentages for toothpaste use (first item) are based on the total sample (*N* = 203). All subsequent items were asked only of children reported to use toothpaste and are based on *n* = 171 toothpaste users.

^a^Multiple responses permitted.

Parents reported that 145 (71.4%) of children used toothpaste every time they brushed, 26 (12.8%) used toothpaste but not every time, 23 (11.3%) did not use toothpaste and 9 (4.4%) had not yet started brushing (Table 1). Almost all children (99.4%) had their own toothpaste. Parents reported using toothpaste to prevent dental caries (71.9%), prevent colouring/staining (52%), as part of a daily routine (39.8%), prevent gum diseases (36.3%) and provide fresh breath (28.7%). About 70.2% of children started to use toothpaste between 1 and 3 years old. More than half of the parents (59.1%) put toothpaste on their children’s toothbrush themselves, and 64.9% used less than 1/3 of the brush length. The distribution of post‐brushing behaviour was: 65.3% rinsing with plenty of water and spitting out, 22.4% swallowing toothpaste while brushing and 12.4% spitting out without rinsing.

Among the 171 children who used toothpaste, regarding brand selection, most parents considered the contents of the toothpaste (87.1%), while only 8.8% reported preferring toothpaste for containing fluoride. Nearly half of the parents (47.6%) chose the brand due to being organic/herbal, followed by dentist recommendation (30%), information on the package (24.1%) and accessibility (15.3%). Some parents reported in writing reasons including being safe to swallow, not containing SLS, fluoride, paraben, titanium dioxide, and glucose. The most popular brand chosen was a fluoride‐free product (29.9%), followed by fluoridated brands (15.2%, 11.4% and 11.4% respectively). About 13% could not remember the brand name (Figure 1).

**Figure 1:**
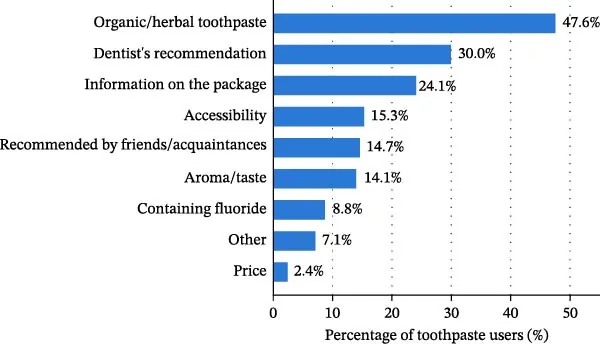
Reasons for toothpaste brand selection (*N* = 171, multiple responses permitted).

The key associations with socio‐demographic variables are presented in Table 2. Consistent toothpaste usage was significantly associated with parent age (*p*  < 0.001): only 25.0% of parents under 29 years reported consistent use, compared to 72%–78% among parents aged 30 and over. Parental education was also significantly associated with toothpaste usage (*p* = 0.019). The time to commence toothpaste usage differed significantly by number of children (*p* = 0.036): 80.0% of single‐child parents started between ages 1–3 years, compared to 59.4% of parents with two children. A significant difference was observed between mothers and fathers regarding reasons for toothpaste usage (*p* = 0.024): more fathers cited gum disease prevention (59.3%) than mothers (30.9%). Fathers also more frequently cited friend recommendations as a brand selection reason compared to mothers (34.6% vs. 9.6%; *p* = 0.011). Post‐brushing swallowing was significantly more prevalent in children under 2 years (61.5% vs. 19.1%; *p* = 0.002) and in single‐child families (32.9% vs. 11.1%; *p* = 0.020) (Table 2).

**Table 2:** Significant associations between toothpaste‐related practices and socio‐demographic variables.

Variable	Parent age (%)				Education (%)			Number of children (%)		*p*
	<29	30–34	35–39	≥40	HS≤	Uni	≥Mast	1	2	
Toothpaste usage—every time	25.0^a^	72.1^b^	77.6^b^	74.0^b^	58.3	73.6	75.5	—	—	< 0.001^1^/0.019^3^
Time to commence toothpaste—1–3 year (%)	—	—	—	—	—	—	—	80.0^a^	59.4^b^	0.036^2^
Swallows toothpaste—<2 year: 61.5%; 2–6 year: 19.1%	—	—	—	—	—	—	—	32.9^a^	11.1^b^	0.002^1^/0.020^2^
Gum disease as reason—mother: 30.9%; father: 59.3%	—	—	—	—	—	—	—	—	—	0.024^4^
Friend rec. as brand reason—mother: 9.6%; father: 34.6%	—	—	—	—	—	—	—	—	—	0.011^4^

*Note:* a–b: Groups sharing the same superscript letter do not differ significantly (*Z* test with Bonferroni correction). HS≤, high school or less; ≥Mast, master’s or doctorate; Uni, university/college.

^1^Child age group comparison.

^2^Number of children comparison.

^3^Education level comparison.

^4^Mother vs. father comparison.

Multivariable logistic regression models, adjusting simultaneously for parental age, education, number of children, child age group and child sex, are presented in Table 3. Consistent toothpaste usage was significantly associated with parental age (aOR 11.10–17.51 for parents aged 30 years and older compared with those under 30; *p*  < 0.001) and with child age group (aOR 14.64 for children aged 2–6 years versus under 2 years; *p*  < 0.001). Early introduction of toothpaste was significantly less likely among children aged 2–6 years than those under 2 years (aOR 0.14; *p* = 0.005). Post‐brushing swallowing of toothpaste was significantly associated with having two or more children (aOR 3.54; *p* = 0.008) and with child age group 2–6 years (aOR 7.82; *p* = 0.002). The overall models were statistically significant for consistent toothpaste usage and for swallowing behaviour (likelihood ratio *p*  < 0.001 and *p* = 0.002, respectively), but not for early introduction, who applied the toothpaste, or amount used. Sensitivity analyses using Firth’s penalised logistic regression, conducted because of small cell counts in some subgroups, yielded consistent estimates (Table 4).

**Table 3:** Multivariable logistic regression models for toothpaste‐related practices (adjusted for parental age, education, number of children, child age group, and child sex; five separate models, one per outcome).

Outcome	Variable	Category	aOR	95% CI	*p*
Consistent toothpaste use (Q6) *n* = 190, appropriate = 142	Parent age	<30 years	1.00 (ref.)	—	—
		30–34 years	**17.51**	**4.05–75.65**	**<0.001**
		35–39 years	**15.69**	**3.81–64.59**	**<0.001**
		≥40 years	**11.10**	**2.66–46.20**	**<0.001**
	Education level	High school or less	1.00 (ref.)	—	—
		University	2.15	0.75–6.21	0.156
		Postgraduate	1.98	0.60–6.53	0.260
	Number of children	1 child	1.00 (ref.)	—	—
		≥2 children	1.03	0.44–2.38	0.948
	Child age group	<2 years	1.00 (ref.)	—	—
		2–6 years	**14.64**	**5.25–40.78**	**<0.00** **1**
	Child sex	Female	1.00 (ref.)	—	—
		Male	0.85	0.39–1.88	0.692

Early introduction of toothpaste (Q9) *n* = 167, appropriate = 20	Parent age	<30 years	1.00 (ref.)	—	—
		30–34 years	0.25	0.03–1.84	0.174
		35–39 years	0.40	0.06–2.58	0.337
		≥40 years	0.56	0.09–3.49	0.539
	Education level	High school or less	1.00 (ref.)	—	—
		University	1.40	0.34–5.76	0.645
		Postgraduate	0.85	0.17–4.34	0.843
	Number of children	1 child	1.00 (ref.)	—	—
		≥2 children	2.53	0.84–7.61	0.100
	Child age group	<2 years	1.00 (ref.)	—	—
		2–6 years	**0.** **14**	**0.04–0.** **56**	**0.005**
	Child sex	Female	1.00 (ref.)	—	—
		Male	0.72	0.26–1.99	0.533

Adult applies toothpaste (Q10) *n* = 160, appropriate = 99	Parent age	<30 years	1.00 (ref.)	—	—
		30–34 years	1.00	0.21–4.72	0.999
		35–39 years	0.91	0.20–4.16	0.899
		≥40 years	0.37	0.08–1.72	0.206
	Education level	High school or less	1.00 (ref.)	—	—
		University	0.53	0.20–1.41	0.207
		Postgraduate	0.93	0.32–2.73	0.896
	Number of children	1 child	1.00 (ref.)	—	—
		≥2 children	0.89	0.43–1.81	0.740
	Child age group	<2 years	1.00 (ref.)	—	—
		2–6 years	0.28	0.06–1.38	0.119
	Child sex	Female	1.00 (ref.)	—	—
		Male	1.01	0.51–1.97	0.985

Appropriate amount of toothpaste (Q11) *n* = 167, appropriate = 110	Parent age	<30 years	1.00 (ref.)	—	—
		30–34 years	0.41	0.07–2.26	0.304
		35–39 years	0.75	0.14–4.09	0.736
		≥40 years	0.56	0.10–3.10	0.507
	Education level	High school or less	1.00 (ref.)	—	—
		University	0.73	0.27–1.96	0.532
		Postgraduate	0.37	0.13–1.06	0.065
	Number of children	1 child	1.00 (ref.)	—	—
		≥2 children	1.14	0.56–2.30	0.721
	Child age group	<2 years	1.00 (ref.)	—	—
		2–6 years	0.79	0.22–2.75	0.707
	Child sex	Female	1.00 (ref.)	—	—
		Male	1.06	0.55–2.06	0.857

Does not swallow toothpaste (Q12) *n* = 166, appropriate = 128	Parent age	<30 years	1.00 (ref.)	—	—
		30–34 years	0.51	0.05–4.87	0.558
		35–39 years	0.71	0.07–6.81	0.767
		≥40 years	0.72	0.07–7.32	0.779
	Education level	High school or less	1.00 (ref.)	—	—
		University	0.46	0.11–1.89	0.284
		Postgraduate	0.32	0.07–1.42	0.134
	Number of children	1 child	1.00 (ref.)	—	—
		≥2 children	**3.54**	**1.40–8.93**	**0.008**
	Child age group	<2 years	1.00 (ref.)	—	—
		2–6 years	**7.82**	**2.18–28.07**	**0.0** **02**
	Child sex	Female	1.00 (ref.)	—	—
		Male	1.21	0.54–2.69	0.644

*Note:* Reference categories: parent age <30 years, education high school or less, 1 child, child age <2 years, child sex female. Bold values indicate *p* < 0.05.

Abbreviations: aOR, adjusted odds ratio; CI, confidence interval.

**Table 4:** Sensitivity analysis using Firth’s penalised logistic regression, conducted because of small cell counts in some subgroups (same models and reference categories as Table 3).

Outcome	Variable	Category	aOR	95% CI	*p*
Consistent toothpaste use (Q6) *n* = 190, appropriate = 142	Parent age	<30 years	1.00 (ref.)	—	—
		30–34 years	**14.21**	**3.78–62.46**	**<0.001**
		35–39 years	**12.90**	**3.57–54.04**	**<0.001**
		≥40 years	**9.29**	**2.54–39.36**	**<0.001**
	Education level	High school or less	1.00 (ref.)	—	—
		University	2.10	0.74–5.81	0.160
		Postgraduate	1.93	0.61–6.13	0.264
	Number of children	1 child	1.00 (ref.)	—	—
		≥2 children	1.02	0.46–2.32	0.953
	Child age group	<2 years	1.00 (ref.)	—	—
		2–6 years	**12.29**	**4.79–34.24**	**<0.001**
	Child sex	Female	1.00 (ref.)	—	—
		Male	0.86	0.40–1.85	0.697

Early introduction of toothpaste (Q9) *n* = 167, appropriate = 20	Parent age	<30 years	1.00 (ref.)	—	—
		30–34 years	0.26	0.04–1.81	0.160
		35–39 years	0.39	0.08–2.50	0.292
		≥40 years	0.53	0.11–3.32	0.464
	Education level	High school or less	1.00 (ref.)	—	—
		University	1.26	0.37–5.40	0.730
		Postgraduate	0.83	0.18–4.08	0.806
	Number of children	1 child	1.00 (ref.)	—	—
		≥2 children	2.29	0.83–6.88	0.109
	Child age group	<2 years	1.00 (ref.)	—	—
		2–6 years	**0.** **16**	**0.05–0.** **59**	**0.007**
	Child sex	Female	1.00 (ref.)	—	—
		Male	0.76	0.28–1.96	0.566

Adult applies toothpaste (Q10) *n* = 160, appropriate = 99	Parent age	<30 years	1.00 (ref.)	—	—
		30–34 years	1.05	0.22–4.38	0.944
		35–39 years	0.96	0.21–3.87	0.959
		≥40 years	0.41	0.09–1.67	0.219
	Education level	High school or less	1.00 (ref.)	—	—
		University	0.56	0.21–1.41	0.222
		Postgraduate	0.95	0.33–2.65	0.915
	Number of children	1 child	1.00 (ref.)	—	—
		≥2 children	0.89	0.45–1.79	0.740
	Child age group	<2 years	1.00 (ref.)	—	—
		2–6 years	0.35	0.06–1.27	0.116
	Child sex	Female	1.00 (ref.)	—	—
		Male	1.00	0.52–1.94	0.997

Appropriate amount of toothpaste (Q11) *n* = 167, appropriate = 110	Parent age	<30 years	1.00 (ref.)	—	—
		30–34 years	0.48	0.08–2.09	0.338
		35–39 years	0.85	0.15–3.69	0.839
		≥40 years	0.65	0.11–2.82	0.578
	Education level	High school or less	1.00 (ref.)	—	—
		University	0.76	0.28–1.91	0.568
		Postgraduate	0.39	0.13–1.07	0.068
	Number of children	1 child	1.00 (ref.)	—	—
		≥2 children	1.13	0.57–2.24	0.728
	Child age group	<2 years	1.00 (ref.)	—	—
		2–6 years	0.84	0.23–2.61	0.767
	Child sex	Female	1.00 (ref.)	—	—
		Male	1.06	0.56–2.02	0.858

Does not swallow toothpaste (Q12) *n* = 166, appropriate = 128	Parent age	<30 years	1.00 (ref.)	—	—
		30–34 years	0.69	0.07–3.92	0.697
		35–39 years	0.94	0.09–5.41	0.951
		≥40 years	0.94	0.09–5.79	0.952
	Education level	High school or less	1.00 (ref.)	—	—
		University	0.53	0.12–1.78	0.321
		Postgraduate	0.37	0.08–1.37	0.142
	Number of children	1 child	1.00 (ref.)	—	—
		≥2 children	**3.19**	**1.37–8.12**	**0.007**
	Child age group	<2 years	1.00 (ref.)	—	—
		2–6 years	**6.68**	**2.07–23.71**	**0.00** **2**
	Child sex	Female	1.00 (ref.)	—	—
		Male	1.19	0.55–2.61	0.653

*Note:* Reference categories as in Table 3. Bold values indicate *p* < 0.05. aOR, adjusted odds ratio (Firth‐penalised estimate); CI, profile‐likelihood confidence interval.

## 4. Discussion

This cross‐sectional questionnaire study was conducted among 203 parents of children aged under 6‐year‐old, attending paediatric outpatient departments in Istanbul. Criteria for toothpaste selection, amount of toothpaste usage and after‐brushing behaviour were used to understand parents’ attitudes toward toothpaste utilisation in children.

The prevalence of toothpaste usage was high (84.2%), consistent with the findings of Jain et al. [18]. Children aged 2–6 years showed significantly higher rates of consistent toothpaste usage (82%) than those under 2 years (22.2%). A significant association was observed between the parental educational level and toothpaste usage, with higher educational levels associated with more consistent use. These data may reflect generational differences or a need for greater oral hygiene knowledge among younger parents. In the multivariable model, parental age remained an independent predictor of consistent toothpaste use (aOR 11.10–17.51, *p*  < 0.001), whereas parental education level, which was significantly associated with toothpaste use in the bivariate analysis (*p* = 0.019), lost statistical significance after adjustment (*p* = 0.156–0.260). This suggests that the bivariate association between education and toothpaste use may be at least partly explained by parental age rather than representing an independent effect of education.

In the present study, most parents reported using toothpaste to prevent dental caries, consistent with studies by Pranno et al. [19] and Shirke and Katre [17]. Relatively more fathers chose gum disease prevention as their reason than mothers. Considering that the prevalence of gingivitis in preschool children is very low, fathers may have unconsciously stated their reasons for their own toothpaste.

Almost all children (99.4%) used children’s toothpaste, separated from their parents. In another study in Eskişehir, Türkiye, by Jahandideh and Tüloğlu [20], 67.4% of children used children’s toothpaste. This discrepancy is likely attributable to socio‐demographic differences between the study populations.

Using toothpaste immediately after eruption of deciduous teeth helps prevent ECC [21]. In the current investigation, only 12.3% of children started using toothpaste immediately after the first tooth eruption; most began between ages 1–3 years (70.2%). Similarly, Bennadi et al. [12] determined that children started toothbrushing with toothpaste more than a year after eruption. The time to commence toothpaste usage was significantly associated with the number of children in the family (*p* = 0.036), likely attributable to differences in parental oral health knowledge and beliefs.

Younger children, particularly those under five, have inadequate swallowing and expectorating reflexes and are more likely to ingest toothpaste while toothbrushing [7]. In the current investigation, more than half of parents (59.1%) reported putting toothpaste on their children’s toothbrushes themselves, and most reported using less than 1/3 of the length of the brush (64.9%), as recommended. A notable proportion of children were reported to swallow toothpaste while brushing (22.4%), consistent with results of Bennadi et al. [12], Sultan et al. [13] and Agboola et al. [22]. Children aged under 6 years should be encouraged to spit out the toothpaste without rinsing to increase the effectiveness of fluoridated toothpaste.

While 87.1% of parents considered the contents of toothpaste when choosing it, only 8.8% specifically mentioned fluoride. Nearly half chose their brand based on organic or herbal ingredients, and the most popular brand chosen was a fluoride‐free product. This pattern likely reflects a broader cultural concern about fluoride in Türkiye, possibly fuelled by social media narratives. Fluoride‐free toothpastes offer no cariostatic benefit, and their use instead of fluoridated alternatives represents a lost opportunity for one of the most cost‐effective preventive interventions available [8]. Dental professionals should proactively communicate the safety and efficacy of age‐appropriate fluoridated toothpaste. Fathers more frequently cited friend recommendations as a brand selection reason compared to mothers, consistent with differences in health information‐seeking behaviour between genders.

These findings have several practical implications for paediatric dentists and paediatricians. Parents should be counselled to begin toothpaste use as soon as the first primary tooth erupts, rather than delaying until later, and to supervise brushing and toothpaste application until children develop adequate spitting ability. Given the high preference for fluoride‐free products, healthcare providers should discuss the safety and cariostatic benefit of age‐appropriate fluoridated toothpaste at routine visits, particularly for younger and less experienced parents. Reinforcing these messages during paediatric and dental check‐ups could improve the consistency of parental toothpaste‐related practices.

This study has several strengths. The questionnaire was developed from previously validated instruments and evaluated by a panel of seven paediatric dentistry experts for content validity before administration. It captured multiple dimensions of toothpaste‐related behaviour, including timing of introduction, amount used, post‐brushing behaviour and brand‐selection criteria, rather than a single usage measure. The sample size met the pre‐specified power analysis requirement, and multivariable regression was used to account for potential confounding among socio‐demographic variables.

### 4.1. Limitations

The questionnaire was self‐reported, which might be subject to recall error and reporting bias. The limited sample size and recruitment from two localised hospitals with a relatively high socio‐economic level limit generalisation to the broader population. Clinical outcomes such as dental caries experience were not assessed in this study, and therefore, the relationship between toothpaste‐related practices and disease status could not be determined. Further research on more extensive and diverse populations is needed for a better understanding of parents’ toothpaste‐related attitudes and practices.

## 5. Conclusions

Despite high overall rates of toothpaste usage amongst preschool children in Istanbul, this study identified critical gaps: delayed introduction, high rates of toothpaste swallowing among younger children and a striking preference for fluoride‐free toothpastes driven by organic/herbal ingredient concerns. Only 8.8% of parents cited fluoride content as a consideration when selecting toothpaste, and the most popular brand chosen was a fluoride‐free product. It would be beneficial for parents to have more information about the importance of using fluoridated toothpaste at an early age, the correct amount and appropriate post‐brushing behaviours. Dental professionals should help and motivate parents to use age‐appropriate fluoridated toothpaste and properly supervise their children’s toothbrushing habits from the eruption of the first deciduous tooth.

## Author Contributions

**Melis Unsal-Caliskaner:** conceptualisation (equal), data curation (lead), formal analysis (lead), investigation (lead), methodology (equal), project administration (lead), visualisation (lead), writing – original draft (lead), writing – review and editing (equal). **Zeynep Seda Uyan**: investigation (supporting), resources (supporting), writing – review and editing (supporting). **S** **enem Selvi Kuvvetli**: conceptualisation (equal), methodology (equal), supervision (lead), validation (lead), writing – review and editing (equal).

## Funding

This study did not receive any specific funding.

## Disclosure

All authors have read and approved the final version of the manuscript. Melis Unsal‐Caliskaner (corresponding author) had full access to all of the data in this study and takes complete responsibility for the integrity of the data and the accuracy of the data analysis.

## Ethics Statement

Ethical approval was granted by the Yeditepe University Non‐Interventional Clinical Research Ethics Committee (date: 15.03.2022/Number 202202162). Written informed consent was obtained from all participants.

## Conflicts of Interest

The authors declare no conflicts of interest.

## Supporting Information

Additional supporting information can be found online in the Supporting Information section.

## Supporting information


**Supporting Information** Dataset S1: Raw survey data supporting the findings of this study, openly available in Mendeley Data at https://doi.org/10.17632/mhhprrnr8f.1.

## Data Availability

The authors confirm that the data supporting the findings of this study are available within the article and/or its Supporting Information.
