# Supplementary material for: Toothpaste‐Related Attitudes and Practices of Parents of Preschool Children in Istanbul, Türkiye: A Cross‐Sectional Study
Source: Int J Dent. 2026 Oct 30;2026:8522497. doi: 10.1155/ijod/8522497 (PMC13720516; doi:10.1155/ijod/8522497)
Supplement: Supplementary file 1 [file IJOD-2026-8522497-s001.docx]

**Questionnaire — Toothpaste-Related Attitudes and Practices of Parents of Preschool Children**

**Part 1: Sociodemographic Information**

1. Please state your age.

………………………………………

2. Please indicate your education level.

a) Primary school

b) Secondary school

c) High school

d) Associate degree (vocational school)

e) University (Bachelor’s)

f) Master’s degree

g) Doctorate

3. Please state your relationship to the child.

a) Mother

b) Father

c) Other …

4. How many children do you have? Please specify.

……………………………………………

5. Please state the date of birth of the child you brought to the paediatric examination.

……………………………………………

6. Please indicate the sex of your child.

a) Female

b) Male

**Part 2: Toothpaste-Related Attitudes and Practices**

1. Is toothpaste used when your child’s teeth are brushed at home?

a) Every time

b) Used, but not every time

c) Toothpaste is not used

*If your answer is (a) or (b), please continue with the questions below.*

2. For what purpose do you use toothpaste when brushing your child’s teeth? (You may select more than one option.)

a) To prevent dental caries

b) To prevent yellowing/staining of teeth

c) To prevent gum diseases

d) To provide fresh breath/prevent bad breath

e) As part of the daily routine

f) Other, please specify …

3. Does your child have their own toothpaste, separate from the parents?

a) Yes

b) No

4. When did you start using toothpaste for your child?

a) As soon as the first deciduous tooth erupted

b) Between 1–3 years of age

c) After 3 years of age

5. Who places the toothpaste on your child’s toothbrush?

a) The child themselves

b) The mother or another adult

6. Which of the following best corresponds to the amount of toothpaste used when your child’s teeth are brushed? Please indicate.

a) Less than 1/3 of the brush

b) Less than 2/3 of the brush

c) The full length of the brush

d) Other, please specify …

7. What does your child do immediately after brushing with toothpaste?

a) Rinses with plenty of water and spits

b) Spits without rinsing with water

c) Swallows the toothpaste during brushing

8. What do you pay attention to when choosing toothpaste for your child? (You may select more than one option.)

a) The content of the toothpaste

b) The flavour/aroma of the toothpaste

c) The images on the toothpaste packaging

d) The price of the toothpaste

e) Other, please specify …

9. Please state the toothpaste brand you currently use for your child.

………………………………

10. What is your reason for choosing this brand? (You may select more than one option.)

a) Dentist’s recommendation

b) Friend/acquaintance recommendation

c) Price

d) Accessibility

e) Information on the packaging

f) Flavour/taste

g) Being organic/herbal

h) Containing fluoride

i) Other, please specify …
